# The expanding role of Nogo-B in hypertension: linking kidney physiology, endothelial function and inflammation

**DOI:** 10.1097/HJH.0000000000004239

**Published:** 2026-01-14

**Authors:** Filipy Borghi, Yannan Jiang, Luigi Gnudi

**Affiliations:** a School of Cardiovascular and Metabolic Medicine & Science, section Vascular Biology and Inflammation, British Heart Foundation Centre of Research Excellence, King's College London; b Department of Diabetes and Endocrinology, Guy's and St Thomas’ Hospital, London, UK

**Keywords:** endothelial function, hypertension, Nogo-B, renal sodium transport, vascular homeostasis

## Abstract

Although Nogo-B has been studied in neural development and vascular biology, its integrative role across renal, vascular and inflammatory pathways in hypertension has not yet been systematically reviewed. Here, we synthesize emerging evidence positioning Nogo-B as a central modulator of blood pressure control. Its expression in the aldosterone-sensitive distal nephron and vascular endothelium suggests a central role in electrolyte and fluid balance, as well as vascular physiology. By bridging insights from the renal, vascular and immune systems, we position Nogo-B as an emerging contributor to blood pressure regulation and highlight its potential both as a biomarker for vascular dysfunction and as a therapeutic target in salt-sensitive and treatment-resistant hypertension

## INTRODUCTION

Hypertension is a leading contributor to global cardiovascular disease, characterized by abnormalities in renal sodium transport, endothelial dysfunction and immune dysregulation [1,2]. Despite advances in understanding its pathophysiology, many molecular mechanisms involved remain unclear. Recent genetic studies have highlighted new candidate genes, including the *RTN4* locus, which reached genome-wide significance in a hypertensive population [3]. This finding has drawn attention to the reticulon family of proteins and their potential role in blood pressure regulation.

Reticulons (RTNs) are a conserved family of membrane-associated proteins critical for shaping the endoplasmic reticulum (ER) and maintaining cellular structure in eukaryotic cells [4]. The family includes four members: RTN1, RTN2, RTN3 and RTN4, which predominantly localize to the ER and regulate its membrane curvature, favouring a tubular over a sheet-like structure [5]. RTN4, also known as Nogo (neurite outgrowth inhibitor), has attracted significant interest due to its inhibitory role in neuronal regeneration. It exists as three isoforms: Nogo-A, Nogo-B and Nogo-C, each with distinct tissue distributions and physiological functions. Nogo-A predominates in the central nervous system and restricts axonal outgrowth [6], while Nogo-C is mainly expressed in skeletal muscle, impairing peripheral nerve regeneration [7]. In contrast, Nogo-B is more broadly expressed, particularly in the kidney, vasculature and inflammatory cells, and exerts functions outside the nervous system [8].

The association between RTN4 and hypertension has stimulated growing interest in Nogo-B, which may play a role in key regulatory systems relevant to blood pressure control. Beyond its established role in neural development, Nogo-B is now recognized for its potential role in controlling electrolyte and fluid balance in aldosterone-sensitive regions of the kidney, where it is highly expressed [9], for its contribution to vascular tone regulation [8], and for its involvement in immune signalling, particularly the upregulation of interleukin-6 (IL-6) [10]. These findings place Nogo-B at the intersection of three major domains involved in the pathogenesis of hypertension: renal sodium handling, vascular function and chronic low-grade inflammation [11,12].

Mechanistically, Nogo-B may exert its effects on blood pressure regulation via several pathways. It interacts with multiple molecular partners, including the Nogo-B receptor (NgBR) and Nogo receptor 1 (NgR1), and also functions independently of membrane-bound receptors by inhibiting serine palmitoyl transferase (SPT), the rate-limiting enzyme in sphingolipid biosynthesis, which mediates downstream signalling pathways affecting endothelial function, ion transport and immune regulation [8,13]. In doing so, Nogo-B modulates levels of sphingosine-1-phosphate (S1P), a lipid mediator that plays essential roles in endothelial barrier function, vascular tone, immune cell trafficking and epithelial sodium transporter regulation [14–16]. While Nogo-B's contributions to vascular biology are increasingly recognized, its full range of actions across renal, vascular and immune systems, and their integration in blood pressure control, remains incompletely understood.

Given the close functional interplay between the vascular and renal systems and the pivotal role of immune activation in hypertension, understanding Nogo-B's integrative functions may provide important insights into novel regulatory mechanisms in blood pressure control. This review synthesizes current knowledge on Nogo-B's role in kidney sodium handling, endothelial biology and inflammation, and explores its potential as a therapeutic target in hypertension and related cardiovascular disorders.

## NOGO-B: MOLECULAR MECHANISMS AND CELLULAR TARGETS

Nogo-B plays a central role in regulating ER structure and dynamics, suggesting broader cellular functions than those typically ascribed to reticulon proteins [17]. Unlike Nogo-A, which is predominantly restricted to the central nervous system, Nogo-B is widely expressed in peripheral tissues, including vascular endothelial cells, smooth muscle, liver, lungs and kidney, supporting its involvement in vascular homeostasis, immune regulation and organ-specific physiological processes [17,18].

**FIGURE 1:**
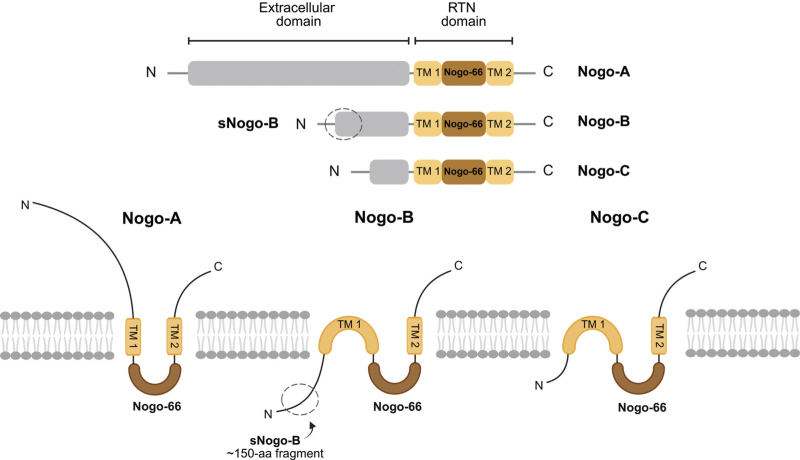
Domain organization of Nogo isoforms. Schematic representation of Nogo-A, Nogo-B and Nogo-C. All share a conserved reticulon (RTN) domain with two transmembrane regions (TM1 and TM2) flanking the Nogo-66 loop. Nogo-A contains a long N-terminal extension critical for neurite outgrowth inhibition. Nogo-B has a unique N-terminal domain and generates a soluble fragment (sNogo-B) with signalling potential. Nogo-C is a truncated isoform mainly expressed in muscle.

Structurally, the differences between Nogo isoforms underlie their distinct biological roles (Fig. 1). Nogo-A exerts its effects through its N-terminal domain and extracellular loop, which are critical for its inhibitory actions on axonal growth [19,20]. In contrast, Nogo-B exerts its biological effects through interactions with two major receptors: NgBR and NgR1, and by inhibiting SPT [8,16,21].

NgBR, which binds specifically to the N-terminus of Nogo-B, is predominantly expressed in endothelial cells, where it regulates key functions such as stimulation of migration, proliferation and angiogenesis, processes essential for maintaining vascular integrity and repair [21,22]. Beyond its membrane-bound form, Nogo-B also exists as a soluble isoform (sNogo-B), a ~150–200-amino-acid N-terminal fragment of the full-length protein that circulates in plasma and acts as a signalling molecule with emerging biomarker potential [23,24]. Like its full-length counterpart, sNogo-B promotes endothelial cell migration and proliferation via NgBR and the Akt signalling pathway [21,25].

In parallel, Nogo-B influences cytoskeletal organisation, vesicular trafficking and membrane dynamics. Through NgR-dependent pathways, Nogo-B modulates RhoA activation. This influences F-actin assembly and α-tubulin stability, both essential for endothelial barrier integrity and vesicular transport [26]. Furthermore, Nogo-B contributes to ER morphology by forming dimers or oligomers with partner proteins to maintain the tubular ER network [27]. This structural integrity is particularly important in renal epithelial cells, where coordinated ER function and cytoskeletal stability ensure the localization and function of key ion transporters necessary for sodium reabsorption and blood pressure control [28].

Nogo-B also exerts receptor-independent effects through inhibition of SPT, reducing intracellular S1P levels. S1P signals through five G protein–coupled receptors (S1PR1–5) and is involved in diverse physiological processes, including vascular permeability, immune cell trafficking and regulation of vascular tone [29]. In the kidney, the S1P/S1PR signalling axis plays a significant role in sodium handling, particularly within the collecting duct, where it influences ion transport processes critical for fluid balance and blood pressure regulation [30]. Importantly, reduced S1P availability, such as that caused by Nogo-B-mediated SPT inhibition, may attenuate this signalling pathway, potentially leading to reduced natriuresis and increased susceptibility to salt-sensitive hypertension. This highlights a novel mechanistic link between Nogo-B and renal blood pressure control, potentially mediated by lipid signalling pathways such as S1P/S1PR.

Despite these advances, the direct mechanistic links between Nogo-B and transporter trafficking remain incompletely defined. Specifically, how Nogo-B interfaces with ion channel localization, membrane recycling and posttranslational regulation in epithelial and endothelial cells is yet to be clarified. Addressing these questions is essential for understanding its broader physiological role and for assessing its therapeutic potential in hypertension, CKD and cardiovascular disease.

## NOGO-B AND RENAL SODIUM HANDLING

**FIGURE 2:**
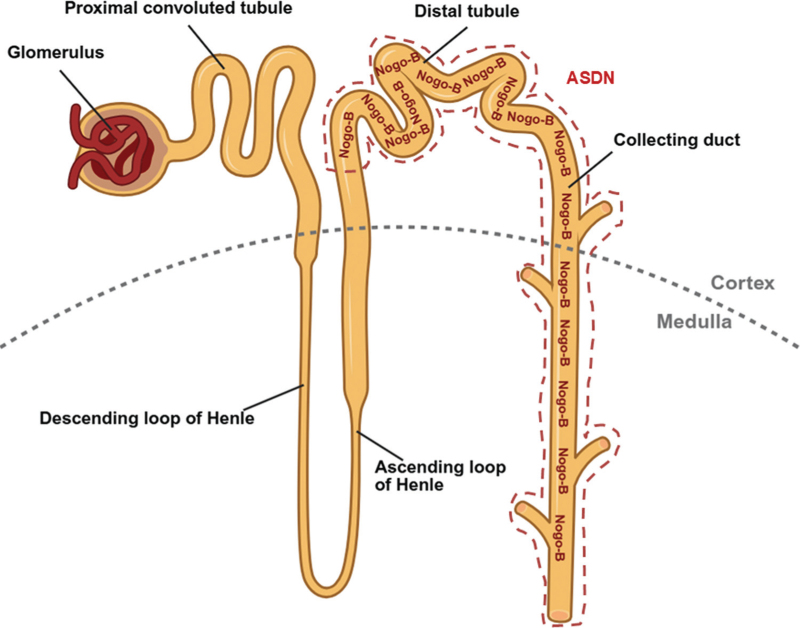
Nogo-B expression in nephron segments. Diagram of nephron architecture highlighting Nogo-B localization (red outline) in the aldosterone-sensitive distal nephron (ASDN), which includes the distal convoluted tubule, connecting tubule and collecting duct. These regions are key for sodium and water reabsorption, implicating Nogo-B in natriuretic control and blood pressure regulation. The dotted grey line separates cortex and medulla.

Nogo-B is increasingly recognized as a regulator of renal physiology, particularly in pathways critical for sodium and fluid balance. It is highly expressed in the aldosterone-sensitive distal nephron (ASDN), a key site for sodium and water regulation [9], which includes the distal convoluted tubule, connecting tubule and collecting duct (Fig. 2). These nephron segments fine-tune sodium reabsorption, potassium secretion and water handling under the control of aldosterone and vasopressin. The localization of Nogo-B in these regions suggests that it may influence electrolyte transport and thereby contribute to blood pressure regulation [2,31–33].

One potential mechanism is the regulation of epithelial sodium channels (ENaC). Proper trafficking, surface expression and degradation of ENaC are central to sodium reabsorption in the distal nephron. Like other reticulon proteins, Nogo-B may affect these processes through interactions with cytoskeletal and vesicular trafficking machinery [8]. Evidence linking Nogo-B to peroxisome proliferator-activated receptor gamma (PPAR-γ) signalling further supports this possibility: PPAR-γ agonists enhance sodium reabsorption and fluid retention [34], while also stimulating Nogo-B expression [35]. This raises the hypothesis that Nogo-B, like PPAR-γ, could amplify ENaC activity and promote sodium retention.

Nogo-B may also intersect with the mineralocorticoid receptor pathway. Aldosterone stimulates sodium reabsorption and potassium excretion by upregulating ENaC expression. Recent findings show that aldosterone also regulates microRNA expression, introducing additional layers of feedback regulation [36]. Although direct links between Nogo-B and these mechanisms remain unclear, it is plausible that Nogo-B modulates aldosterone responses either at the transcriptional level or via posttranslational mechanisms such as phosphorylation or ubiquitination of ion channels.

Beyond direct channel regulation, Nogo-B influences sphingolipid metabolism. The S1P–S1PR1 axis has been directly implicated in natriuretic control: deletion of S1PR1 in collecting duct principal cells promotes sodium retention and worsens salt-induced hypertension [37], whereas S1PR1 activation reduces ENaC activity and improves sodium excretion [38]. In humans, functional antagonism of S1PR1 with agents such as fingolimod or siponimod has been associated with higher blood pressure and cardiovascular risk [39–41]. These findings position Nogo-B as an upstream regulator of this pathway, potentially linking sphingolipid metabolism to renal sodium handling [30,42].

Despite these insights, important uncertainties remain. It is still unclear whether Nogo-B directly modulates ENaC trafficking or acts mainly through sphingolipid signalling. Similarly, the extent to which Nogo-B interacts with aldosterone-driven transcriptional networks is unknown. Conditional knockout models targeting renal epithelial cells will be crucial to dissect the compartment-specific contributions of Nogo-B to sodium reabsorption and salt-sensitive hypertension.

## NOGO-B IN ENDOTHELIAL REGULATION AND VASCULAR TONE

Nogo-B is highly expressed in vascular endothelial cells, where it regulates key processes such as angiogenesis, barrier function and endothelial survival [21]. These effects are largely mediated through the NgBR, which activates the Akt pathway to promote cell proliferation, migration and cytoskeletal organization [43,44]. A soluble isoform, sNogo-B, circulates in plasma and can further stabilize the endothelium by modulating VEGFA/VEGFR2 signalling [45].

Through its interactions with NgBR, Nogo-B supports endothelial integrity. However, its role is not uniformly protective. Since S1PR1 activation promotes endothelial nitric oxide synthase (eNOS) activity and nitric oxide (NO) production [46,47], reduced S1P availability can impair vasodilation and endothelial barrier function [48]. In contrast, Nogo-B deficiency has been associated with enhanced S1PR1 signalling, increased NO production and protection against hypertension-induced endothelial dysfunction [49]. This duality highlights the context-specific nature of Nogo-B's effects [8].

Beyond the endothelium, Nogo-B also influences vascular smooth muscle cells (VSMCs). It inhibits VSMC proliferation and migration, processes that normally drive vascular remodelling in hypertension and atherosclerosis [50]. In this way, Nogo-B plays a dual role: maintaining vascular stability under physiological conditions, while limiting pathological remodelling under stress.

The vascular role of Nogo-B intersects with the renin–angiotensin–aldosterone system (RAAS), a key hormonal axis in blood pressure regulation [51]. RAAS activation drives vasoconstriction and sodium retention through angiotensin II and aldosterone, respectively [52]. In mouse models, Nogo-B deficiency confers resistance to angiotensin II–induced hypertension and vascular inflammation [8,53], suggesting interactions between Nogo-B and endocrine regulators of vascular tone.

How Nogo-B balances its supportive roles in endothelial stability with its inhibitory effects on S1P signalling remains uncertain. Further work is needed to determine whether these apparently conflicting actions are tissue-specific, context-dependent, or influenced by disease state.

## NOGO-B IN VASCULAR INFLAMMATION AND IMMUNE-MEDIATED HYPERTENSION

In addition to its renal and vascular roles, Nogo-B has emerged as a regulator of vascular inflammation and immune activation, both recognized as key drivers of hypertension. Chronic low-grade inflammation contributes to endothelial dysfunction, vascular stiffening and sodium retention [54], and growing evidence places Nogo-B at the intersection of lipid metabolism and cytokine signalling.

One important mechanism is the modulation of interleukin-6 (IL-6). Nogo-B upregulates IL-6 signalling, thereby amplifying inflammatory responses [10,55] that enhance ENaC activity and promote sodium retention in the distal nephron [56]. Elevated IL-6 is a hallmark of experimental and clinical hypertension, and its blockade reduces renal inflammation and blood pressure in animal models [12,13]. Interestingly, Nogo-B may also indirectly suppress IL-6 by reducing S1P availability, since S1P can stimulate IL-6 expression through PI3K/Akt and MEK/ERK pathways [57]. This apparent paradox suggests that Nogo-B's effect on IL-6 may be highly context-dependent, differing by tissue or inflammatory state.

Nogo-B also shapes innate immune responses. In models of lipopolysaccharide (LPS)-induced inflammation, Nogo-B enhances Toll-like receptor (TLR) clustering and NF-κB activation, leading to increased secretion of IL-6, TNF-α and IL-1β [58,59]. Conversely, Nogo-B deficiency reduces TLR signalling and cytokine production [10], whereas Nogo-B overexpression amplifies these responses [16]. These findings indicate that Nogo-B actively drives, rather than passively marks, inflammatory activity.

Another mechanism involves leukocyte recruitment. Nogo-B modulates expression of adhesion molecules such as intercellular adhesion molecule-1 (ICAM-1) and vascular cell adhesion molecule-1 (VCAM-1) on endothelial cells. Its effects appear context-specific: under baseline conditions, loss of Nogo-B increases ICAM-1 expression and leukocyte infiltration, suggesting an anti-inflammatory role [43]. However, in settings of oxidative stress or pressure overload, Nogo-B expression rises and promotes leukocyte adhesion through ROS–p38–NF-κB signalling [60].

The contrasting effects of Nogo-B, protective under physiological conditions yet pro-inflammatory in stress states, remain to be fully explained. Future studies should define how Nogo-B integrates lipid mediator signalling with cytokine and adhesion pathways, and whether its role in immune activation is a potential therapeutic target in salt-sensitive or treatment-resistant hypertension.

## TRANSLATIONAL POTENTIAL AND FUTURE DIRECTIONS

The diverse roles of Nogo-B across renal, vascular and immune systems highlight its potential as both a biomarker and a therapeutic target in hypertension. By integrating molecular mechanisms with translational perspectives, Nogo-B emerges as a promising candidate for intervention in salt-sensitive and treatment-resistant forms of the disease.

In the kidney, Nogo-B's putative regulation of ENaC trafficking and sphingolipid metabolism suggests opportunities for modulating sodium reabsorption and fluid balance. Strategies that selectively disrupt Nogo-B's interaction with ER trafficking machinery, or that counteract its stimulatory effect on ENaC activity, could improve natriuresis without the systemic side effects of current diuretics. Given that PPAR-γ agonists upregulate Nogo-B and cause fluid retention [34,35], re-evaluating these therapies in light of Nogo-B biology may provide new insights into drug safety and repurposing.

From a vascular standpoint, interventions that restore S1P–S1PR1 signalling may enhance endothelial nitric oxide production, improve barrier function and reduce arterial stiffness [8,49]. Modulating sNogo-B levels, either pharmacologically or via recombinant approaches, could also promote endothelial repair and angiogenesis in vascular beds vulnerable to hypertensive damage. In this context, Nogo-B represents a mechanistic link between lipid signalling and vascular tone that could be leveraged therapeutically.

Targeting Nogo-B-driven inflammation offers another avenue for intervention. Inhibiting Nogo-B-induced IL-6 production, dampening its amplification of TLR–NF-κB signalling, or modulating leukocyte adhesion may help limit the chronic vascular inflammation that drives hypertensive organ damage. Such approaches may be particularly relevant in patients with inflammatory or salt-sensitive hypertension, where immune dysregulation is central to disease progression.

The translational relevance of Nogo-B is further supported by clinical evidence. Elevated circulating sNogo-B levels have been reported in hypertensive patients, particularly in those with vascular dysfunction [61]. This raises the possibility of using sNogo-B as a biomarker for early disease detection, risk stratification or treatment monitoring. In addition, combining Nogo-B measurements with established biomarkers of renal and vascular injury may improve patient profiling and personalized therapy.

To advance these translational opportunities, several gaps must be addressed. Conditional and cell-specific knockout models, particularly in renal epithelial and vascular endothelial cells, are needed to disentangle compartment-specific effects. Clinical studies should clarify the utility of sNogo-B as a biomarker and assess how it responds to antihypertensive therapies. Exploring Nogo-B's regulation by microRNAs, posttranslational modifications and its interplay with transporter trafficking may also reveal novel pharmacological targets.

**FIGURE 3:**
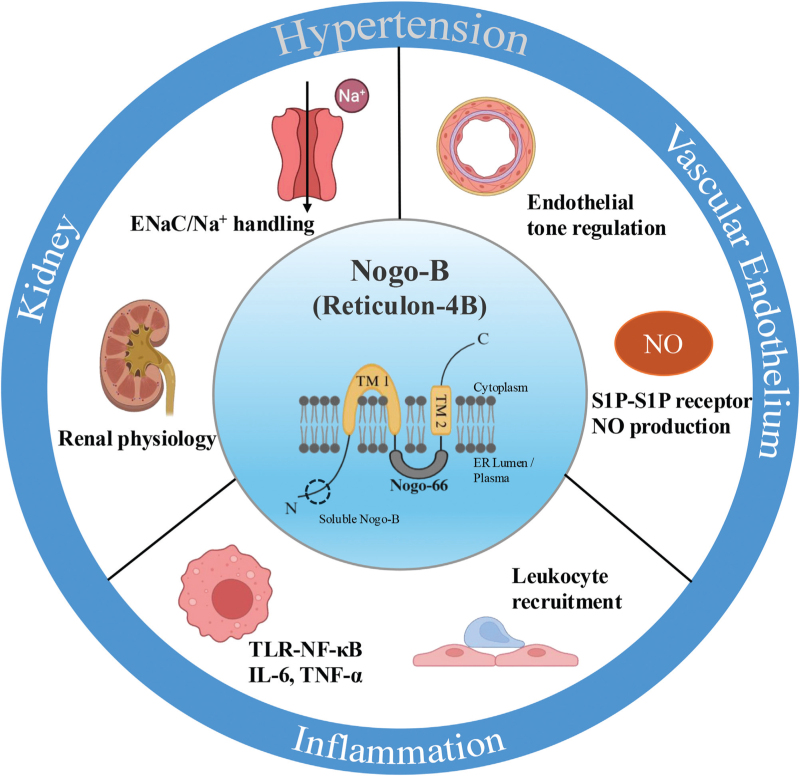
Integrative role of Nogo-B in hypertension. Illustration of the main pathways linking Nogo-B (Reticulon-4B) to blood pressure regulation. In the kidney, Nogo-B modulates ENaC-mediated sodium handling and renal physiology. In the vascular endothelium, it influences endothelial tone and nitric oxide (NO) production via S1P–S1P receptor signalling. In inflammation, it promotes leukocyte recruitment and activation of pro-inflammatory pathways (TLR–NF-κB, IL-6, TNF-α). Together, these actions place Nogo-B at the intersection of renal, vascular and immune mechanisms driving hypertension.

In summary, Nogo-B sits at the convergence of renal sodium handling, vascular function and immune activation (Fig. 3). Its dual role as a molecular regulator and potential clinical marker makes it a compelling focus for future research. Defining and targeting Nogo-B pathways may open new therapeutic avenues for patients with complex or treatment-resistant hypertension, ultimately bridging mechanistic insights with clinical application.

Given the substantial treatment burden of resistant and salt-sensitive hypertension, and the limitations of current therapies in addressing the underlying endothelial dysfunction, Nogo-B-NgBR /-NgR receptor system, and the Nogo-B/S1P-S1PR signalling axes represent clinically relevant targets with multisystem therapeutic potential.

Immediate priorities include validating sNogo-B as a biomarker for patient stratification, elucidating cell-specific contributions through conditional knockout models, and developing selective inhibitors that preserve vascular homeostasis while reducing sodium retention and inflammation. Such mechanistic and translational advances may ultimately enable precision-medicine approaches in hypertension, particularly for populations at highest cardiovascular risk.

## ACKNOWLEDGEMENTS

The authors thank Tarsilo Onuluk for his comments and for reading the manuscript. This work was supported by the British Heart Foundation (PG/24/11441) and Diabetes UK (22/0006394).

**Previous presentations:** The work has not been presented previously in whole or in part.

**Funding sources:** This work was supported by the British Heart Foundation (PG/24/11441) and Diabetes UK (22/0006394).

### Conflicts of interest

There are no conflicts of interest.
